# Pharyngeal-Cervical-Brachial Variant of Guillain-Barré Syndrome in a Patient of COVID-19 Infection

**DOI:** 10.7759/cureus.17945

**Published:** 2021-09-13

**Authors:** Jasneet Randhawa, Harneet S Randhawa, Prabhdeep Toor

**Affiliations:** 1 Medicine, Fortis Escorts Hospital, Amritsar, IND; 2 Radiology, Government Medical College & General Hospital, Baramati, Baramati, IND; 3 Medicine, Bhai Daya Singh Ji Charitable Hospital, Amritsar, IND

**Keywords:** pharyngo-cervical-brachial variant, covid-induced guillian barre syndrome, post covid gbs/atm, guillian barré syndrome, immunological response, neurological autoimmune disorders, systemic autoimmune disease, gbs variant, autoimmune neurology, peripheral motor neuropathy

## Abstract

Guillain-Barré syndrome (GBS) has an annual incidence rate ranging from 0.4 to 1.7 cases per 1,00,000 population. Pharyngeal-cervical-brachial (PCB) variant is an extremely rare variant of GBS (3%), which presents with muscle weakness initially involving the neck, oropharynx, and upper extremities.

GBS often has an infectious inciting event leading to an autoimmune response. There has been an increase in the incidence of GBS during the COVID-19 pandemic, and several case studies have shown an association between the development of GBS and COVID-19 infection. High clinical suspicion is needed to reach a diagnosis. As PCB variant of GBS can have fatal outcomes, a good clinical knowledge of its presentation can allow timely life-saving interventions.

Here, we report a case of GBS with acute onset of neck and respiratory muscle weakness that progressed to upper limb weakness. The patient developed these symptoms two weeks after the onset of cough, fever, and malaise. PCB variant of GBS should always be considered as an important differential diagnosis in any patient presenting with limb weakness and bulbar palsy.

## Introduction

Guillain-Barré syndrome (GBS) is an acute progressive autoimmune polyradiculoneuropathy mostly involving the lower extremity motor nerves and ascending upward. GBS has an infectious preceding event leading to a hyperactive autoimmune response from the immune system against myelin coating of neurons predominantly in motor nerves. The pharyngeal-cervical-brachial (PCB) variant of GBS is interesting because of its unique presentation involving the neck, upper limb, and oropharyngeal muscles. It has a very low incidence and a significant risk of mortality due to the early involvement of respiratory muscles [1,2].

COVID-19 is caused by severe acute respiratory syndrome coronavirus 2 (SARS-CoV-2) virus. COVID-19 often presents with a sore throat, fever, and dry cough. Other complications from a prothrombotic state such as stroke, thromboembolism, etc. are also common in COVID-19 infection. GBS is one of the rare complications associated with COVID-19 infection, and not many cases of PCB variants have been reported [3].

## Case presentation

**Figure 1:**
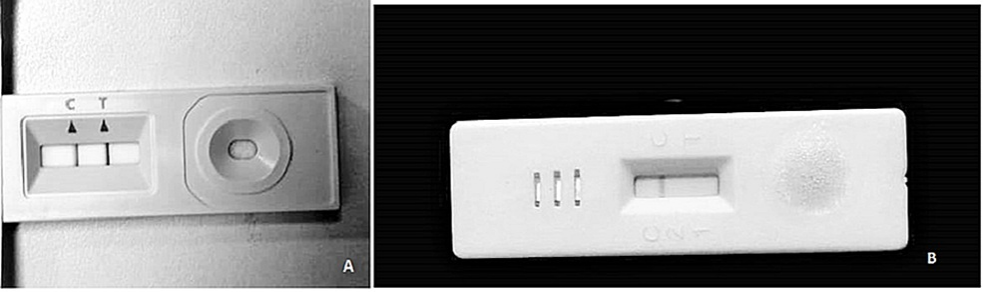
COVID-19 rapid antigen testing kits. (A) Positive test result on the onset of cough and fever 2 weeks ago. (B) Negative test result on admission for difficulty breathing, dysphagia, and upper limb weakness.

A 33-year-old male came to our emergency department with complaints of difficulty in breathing, upper limb weakness, dysphagia, and generalized weakness for two days. Two weeks before the onset of these symptoms, the patient had a cough and fever and was diagnosed with COVID-19 after the SARS-CoV-2 antigen test. Having mild symptoms and no other complications, the patient was advised of home isolation. SARS-CoV-2 antigen testing on admission was negative (Figure 1).

His vitals on admission were within normal limits. SpO2 was 89% on non-invasive ventilation (NIV). The patient was afebrile. On examination, single breath count (SBC) was 18, and power in upper limbs was decreased with absent deep tendon reflexes (DTR). Power in lower limbs was mildly decreased, and DTR was preserved (Table 1).

**Table 1:** Motor examination of the peripheral nervous system (PNS) on admission

	Neck	Shoulder	Upper Limb	Hand Grip	Hip	Lower Limb
Power	2/5	2/5	2/5	70%	4/5	4/5

**Figure 2:**
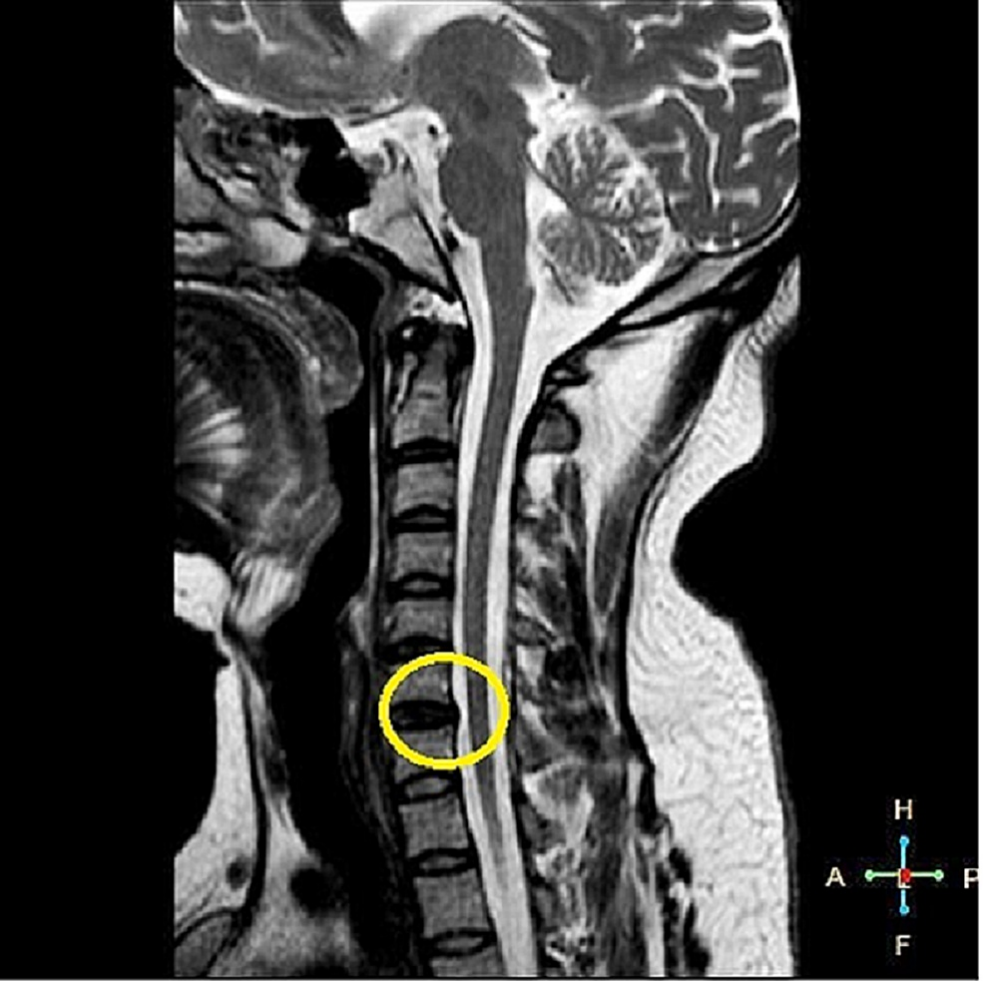
Mid-sagittal T2W image of the cervical spine showing C6-C7 disk desiccation with a mild bulge

MRI of the brain and cervical spine was done. No obvious abnormality or abnormal signal was noted in the brain or spinal cord. C6-C7 disk desiccation with mild bulge and no neural compromise was noted (Figure 2).

Thyroid function tests were within normal limits. Serum antinuclear antibody testing was negative. There was no loss of pain or temperature perception in extremities.

**Figure 3:**
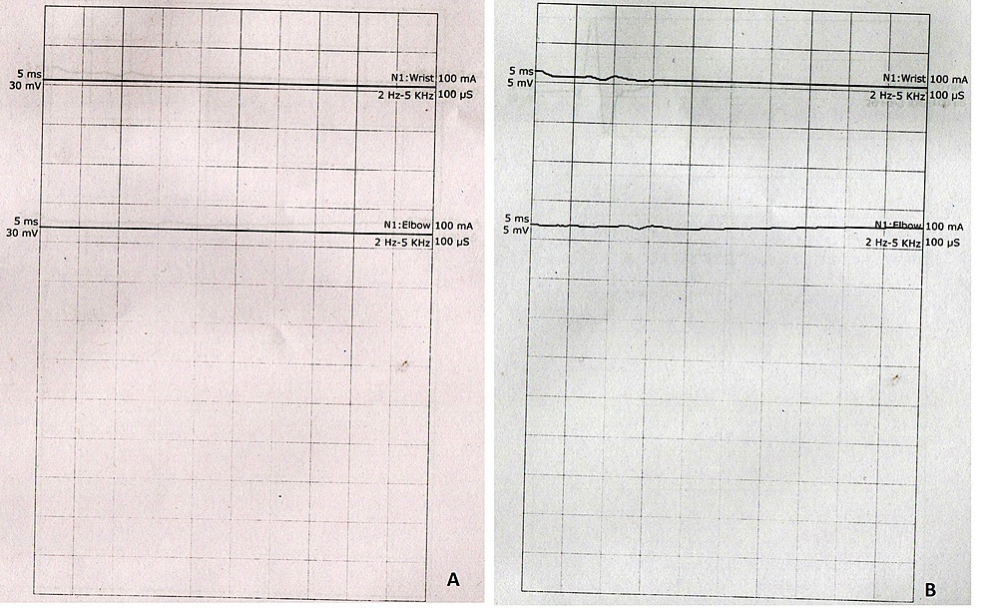
Motor nerve conduction graph of the median nerve at the level of wrist showing an absence of nerve conduction: (A) right median nerve and (B) left median nerve

**Figure 4:**
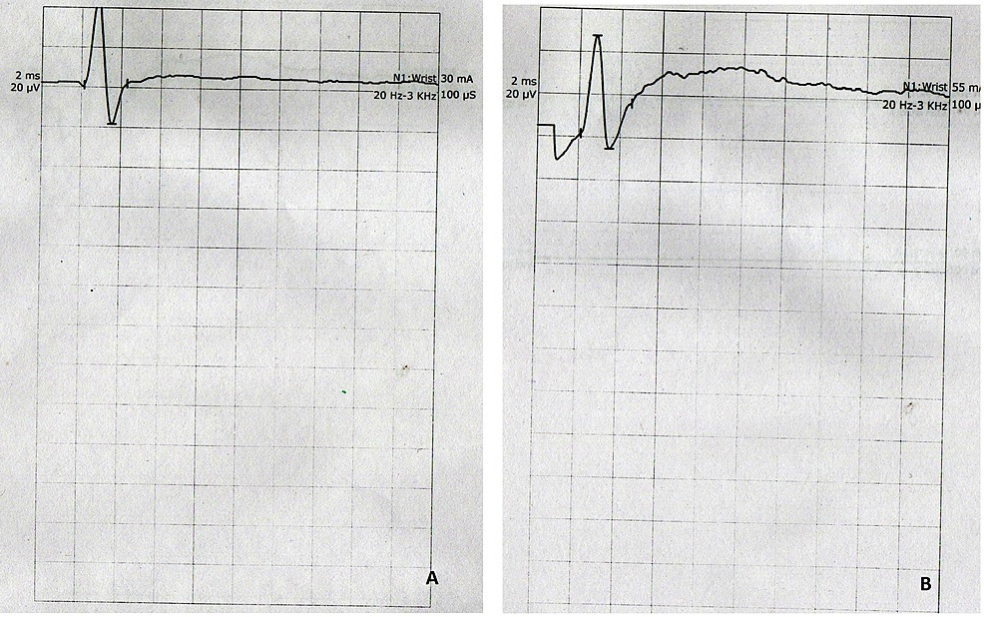
Sensory nerve conduction of the median nerve at the level of wrist showing the presence of nerve conduction to correlate with Figure 3. (A) Left median nerve and (B) right median nerve

Clinical diagnosis of PCB variant of GBS was made, which was confirmed with the nerve conduction study (Tables 2-4). Neurophysiological study of the peripheral nerves showed severe motor neuropathy of upper limbs along with reduced distal compound muscle action potential amplitudes in ulnar, median, tibial, and peroneal nerves bilaterally. Sensory parameters of the median, ulnar, and sural nerves were normal. The F-waves study also confirmed peripheral motor neuropathy (Figures 3, 4).

**Table 2:** Motor nerve conduction (MNC) studies Two electrodes are placed on a motor nerve. An impulse is generated in one and received through another, and the time taken for conduction is calculated. The delay in conduction or complete absence of conduction indicates neuropathy and helps in assessing peripheral neuropathy. The absence of values in this table indicates significant nerve damage leading to the absence of conduction and an inability to test the nerve. Lat 1, Latency 1; LaT 2, Latency 2; AMP, amplitude; Dist, distance; CV, conduction velocity. APB, abductor pollicis brevis; EDB, extensor digitorum brevis; ADM, abductor digiti minimi; EHL, extensor hallucis longus.

Nerve: Median-Lt R-Site: APB					
Stimulation Site	Lat 1 (ms)	Lat 2 (ms)	AMP	Dist (mm)	CV (m/s)
1. Wrist					
2. Elbow					
Nerve: Median-Rt, R-Site: APB					
Stimulation Site	Lat 1 (ms)	Lat 2 (ms)	AMP	Dist (mm)	CV (m/s)
1. Wrist					
2. Elbow					
Nerve: Peroneal-Lt, R-Site: EDB					
Stimulation Site	Lat 1 (ms)	Lat 2 (ms)	AMP	Dist (mm)	CV (m/s)
1. Ankle	5.75		100	70	
2. Knee	13.7		73.7	342	45
Nerve: Peroneal-Rt, R-Site: EDB					
Stimulation Site	Lat 1 (ms)	Lat 2 (ms)	AMP	Dist (mm)	CV (m/s)
1. Ankle	5.8		100	70	
2. Knee	13.6		75	340	48
Nerve Tibial-Lt, R-Site: EHL					
Stimulation Site	Lat 1 (ms)	Lat 2 (ms)	AMP	Dist (mm)	CV (m/s)
1. Ankle	5		100	95	42
2. Popliteal Fossa	12.92		4.18	310	43
Nerve Tibial-Rt, R-Site: EHL					
Stimulation Site	Lat 1 (ms)	Lat 2 (ms)	AMP	Dist (mm)	CV (m/s)
1. Ankle	5.2		100	96	45
2. Popliteal Fossa	12.84		2.6	322	46.1
Nerve: Ulnar-Lt, R-Site: ADM					
Stimulation Site	Lat 1 (ms)	Lat 2 (ms)	AMP	Dist (mm)	CV (m/s)
1. Wrist					
2. Elbow					
Nerve: Ulnar-Rt, R-Site: ADM					
Stimulation Site	Lat 1 (ms)	Lat 2 (ms)	AMP	Dist (mm)	CV (m/s)
1. Wrist					
2. Elbow					

**Table 3:** Sensory nerve conduction (SNC) studies Lat 1, Latency 1; LaT 2, Latency 2; AMP, amplitude; Dist, distance; CV, conduction velocity.

Nerve: Median Wrist-Lt, R-Site					
Stimulation Site	Lat 1 (ms)	Lat 2 (ms)	AMP	Dist (mm)	CV (m/s)
1. Wrist	2.15	4.3	62.6 microV		
Nerve: Median Wrist-Rt, R-Site					
Stimulation Site	Lat 1 (ms)	Lat 2 (ms)	AMP	Dist (mm)	CV (m/s)
1. Wrist	2.1	4.55	52.9 microV	140	66.67
Nerve Sural-Lt, R-Site: Ankle					
Stimulation Site	Lat 1 (ms)	Lat 2 (ms)	AMP	Dist (mm)	CV (m/s)
1. Mid-calf	1.65	4.8	24.6 microV	100	60.61
Nerve Sural-Rt, R-Site: Ankle					
Stimulation Site	Lat 1 (ms)	Lat 2 (ms)	AMP	Dist (mm)	CV (m/s)
1. Mid-calf	1.75	4.7	25.5 microV	100	57.14
2. Popliteal fossa					
Nerve: Ulnar Wrist-Lt, R-Site					
Stimulation Site	Lat 1 (ms)	Lat 2 (ms)	AMP	Dist (mm)	CV (m/s)
1. Wrist	1.85	4.15	62.9 microV	140	75.68
Nerve: Ulnar Wrist-Rt, R-Site					
Stimulation Site	Lat 1 (ms)	Lat 2 (ms)	AMP	Dist (mm)	CV (m/s)
1. Wrist	1.9	4.45	62.9 microV	140	73.68

**Table 4:** F-wave study MNC, Motor nerve conduction; SNC, sensory nerve conduction. (Fmin-M)-lat, F-wave minimum rise-M-wave latency.

Nerve: Median-Lt, R-Site: APB						
M-Lat (mS)	Fmin-Lat (ms)	Fmax-Lat (ms)	Fmean-Lat (ms)	(Fmin-M)-Lat (ms)	Distance (mm)	F-Velocity (m/s)
9.25	23	25	24	13.75		0
Nerve: Median-Rt, R-Site: APB						
M-Lat (mS)	Fmin-Lat (ms)	Fmax-Lat (ms)	Fmean-Lat (ms)	(Fmin-M)-Lat (ms)	Distance (mm)	F-Velocity (m/s)
9.5	23	25	24	13.5		0
Nerve: Tibial-Lt, R-Site: Abductor hallucis						
M-Lat (mS)	Fmin-Lat (ms)	Fmax-Lat (ms)	Fmean-Lat (ms)	(Fmin-M)-Lat (ms)	Distance (mm)	F-Velocity (m/s)
7.5	59.25	67.25	63.25	51.75		0
Nerve: Tibial-Rt, R-Site: Abductor hallucis						
M-Lat (mS)	Fmin-Lat (ms)	Fmax-Lat (ms)	Fmean-Lat (ms)	(Fmin-M)-Lat (ms)	Distance (mm)	F-Velocity (m/s)
7.25	30	32	31	22.75		0

The F-wave study is a pure motor test, which is similar to MNC and SNC. An electrode produces a supra-maximal stimulus that travels backward into the anterior horn cells of the spinal cord and travels back into the motor nerve from there. This test is used to check the proximal segment of the nerve and helps to assess the causes of peripheral neuropathy [4].

After confirming the diagnosis with nerve conduction studies, intravenous immunoglobulin (IVIG) therapy was started. The patient's condition started to improve after 48 hours of treatment onset. As dyspnea improved, the patient was weaned off from NIV on the third day.

The patient was discharged with a prescription of Pregabalin and multivitamins and was advised regular physiotherapy. The patient had weak DTRs, and SBC was 44 on discharge (Table 5).

**Table 5:** Physical examination before discharge from the hospital

	Neck	Shoulder	Upper Limb	Hand Grip	Hip	Lower Limb
Power	3/5	3/5	3/5	80%	4 ± 5	4 ± 5

## Discussion

We present a case of the PCB variant of GBS in a COVID-19-recovered patient. Several infections like *Campylobacter jejuni*, cytomegalovirus (CMV), diphtheria, HIV, etc. have been known to predispose a patient to GBS. Few cases of COVID-19 infection have also resulted in GBS according to recent case studies, and less than a handful have resulted in PCB variant.

Molecular mimicry is the proposed mechanism where the body's immune system makes antibodies against the infectious antigens, and these antibodies cross-react with normal body proteins and molecules leading to immune system activation and damaged body tissues. IgG (anti-GT1a) autoantibodies that react with the neuronal gangliosides are strongly positive in the PCB variant of GBS. These antibodies damage the peripheral nerves and cause a decrease in the conduction of nerve impulses. This causes motor neuropathy. The antibodies preferentially target the motor nerves compared to the sensory nerves and produce the characteristic syndrome [5].

Several documented cases have shown an association between SARS-CoV-2 infection and GBS. Early intervention is needed in the PCB variant of GBS due to a higher risk of mortality compared to GBS. PCB variant of GBS and stroke can have a similar presentation. As there is an increased risk of hypercoagulability with COVID-19 infection, stroke is an important differential to be ruled out first for its effective and timely management. In our case, MRI helped to rule out stroke. The second common differential could be compression neuropathy. MRI of the cervical spine rules out compression neuropathy. Miller-Fisher syndrome (MFS) is a rare variant of GBS with a similar presentation, but lack of ophthalmoplegia which is a characteristic feature of MFS helped exclude this differential. Several endocrine disorders of the thyroid and adrenal glands are associated with peripheral neuropathies, but lack of suggestive symptoms for the same along with rapid development of symptoms in our patient and normal blood work helped rule out this differential diagnosis in our patient [6-8].

GBS has a very rapid progression, and early intervention is always preferred to decrease the severity and to reduce the plateau duration of the disorder. There was an improvement in the administration of intravenous immunoglobulins suggesting a rather manageable form of the syndrome in our patient.

## Conclusions

PCB variant of GBS has several differential diagnoses, and due to its rapid course and high fatality rate, a high clinical suspicion is needed in framing the diagnosis. Knowledge about how a case may present in a clinical setting is important for timely management. As statistical association does not imply causation, more epidemiological data and research are needed to exactly find how the SARS-CoV-2 virus aids in producing this syndrome and if any patient-specific trait increases its probability to cause GBS.
